# Proceedings of the Fourteenth Annual UT- KBRIN Bioinformatics Summit 2015

**DOI:** 10.1186/1471-2105-16-S15-I1

**Published:** 2015-10-23

**Authors:** Eric C Rouchka, Julia H Chariker, Benjamin J Harrison

**Affiliations:** 1 Department of Computer Engineering and Computer Science, University of Louisville, Duthie Center for Engineering, Louisville, KY 40292, USA; 2 Kentucky Biomedical Research Infrastructure (KBRIN) Bioinformatics Core, 522 East Gray Street, Louisville, KY 40292, USA; 3 Department of Psychological and Brain Sciences, University of Louisville, Louisville, KY 40292, USA; 4 Department of Anatomical Sciences and Neurobiology, University of Louisville, Louisville, KY 40292, USA

## 

The University of Tennessee (UT) and the Kentucky Biomedical Research Infrastructure Network (KBRIN) have collaborated over the past fourteen years to share research and educational expertise in bioinformatics. One result is an annual regional summit for researchers, educators and students. The Fourteenth Annual UT- KBRIN Bioinformatics Summit was held at Paris Landing State Park in Buchanan, Tennessee from March 20-22, 2015. A total of 200 participants pre-registered, with 115 from Tennessee, 69 from Kentucky, and the remainder from various states and international locales. Among the registrants were 70 faculty, 65 students, 40 staff, and 23 postdocs. The conference program consisted of a workshop on R and three days of presentations broken into plenary sessions: Where are They Now, Medical Informatics, and Epigenetics. In addition, a poster session with 42 posters was held on Saturday evening.

## Friday workshops

Danny Arends (Humbolt University in Berlin) began the Summit with the workshop “Using R/qtl MQM to improve QTL mapping in inbred crosses.” This workshop covered the use of the free R package R/qtl for mapping and exploring quantitative trait loci (QTL). Included were topics related to Multiple QTL Mapping (MQM), such as handling missing data, using permutations for determining significance thresholds for QTL and QTL hotspots, and visualizations for cis-trans and QTL interaction effects[1,2].

Following this workshop, Dr. Arends presented “CTL Mapping in Inbred Strains.” He covered the topic of correlated traits loci (CTL), a novel method used to correlate differences in expression to regions of the genome[3,4]. CTL detects a number of regions not found with traditional QTL mapping, and therefore increases the number of candidate regions in QTL and genome-wide association studies (GWAS).

## Session I: where are they now

Dan Goldowitz (UBC), one of the original organizers of the Bioinformatics Summit, opened the scientific session with a Bioinformatics Summit Retrospective. As part of this presentation, Dr. Goldowitz discussed the beginnings of the Bioinformatics Summit as part of the University of Tennessee Health Science Center's (UTHSC) Center for Integrative and Translational Genomics (CITG). Throughout this presentation, discussions were made about where former keynote and plenary speakers as well as attendees were, and the current directions of their research.

Ramin Homayouni (University of Memphis) followed with an engaging presentation “From Junior Faculty to Center Director and Entrepreneur” which he renamed “Hindsight is 20/20”. During his presentation, Dr. Homayouni progressed from where he began as a biologist studying the role of individual genes in the nervous system[5-7] to part of the self-proclaimed “Dirty Dozen” at UTHSC which formed the basis of the CITG from which the Bioinformatics Summit originated. Ramin discussed how the Summit has been critical in forming collaborations, leading to the founding of his company, ComputableGenomix, which developed the GeneIndexer software based largely upon text-mining approaches[8-12].

## Session II: medical informatics

Hauke Bartsch (UCSD) kicked off the plenary speaker session with “PING: Pediatric Imaging, Neurocognition, and Genetics.” The PING Data Resource involves 1,400 students aged 3-21 years during childhood and adolescent development[13]. The goal of PING is to research what a normal child looks like during development, and measures information including demographic, developing mental and emotional functions, family history, cognitive abilities (attention and memory), multimodal neuroimaging, and genotype information. PING is a longitudinal study, taking measurements as part of a summer camp experience. The goal is to track “normal” development, including changes in cortical thickness over time, hippocampal volume, and fiber tracts. Specific types of data gathered include age, gender, handedness, height, education, family history, body mass index (height and weight), mini mental state (30 questions), genetic “Europeanness” (based on 550,000 single nucleotide polymorphisms), cortical information (surface area, thickness, and volume), sub-cortical volume, white matter volume, and diffusivity (40 tracts). Among the results of PING include a gyrus-based brain atlas[14], a genetically derived brain atlas[15], and a web portal for exploring and visualizing the PING data, which allows for a “Big Data” hypothesis space[16] by including datasets from a total of 21 different projects.

Joshua Denny (Vanderbilt University) followed with “Harnessing routine clinical care to accelerate genomic discovery …or entering the era of Precision Medicine using EHRs.” This presentation began with an overview of the history of personalized medicine, which goes back to Hippocrates[17], and more recently has been touted by President Obama with the announcement of the new Precision Medicine Initiative[18]. Dr. Denny discussed personalized medicine in the context of Vanderbilt's BioVU[19,20], an opt-out DNA Biobank started in 2007 which contains a cohort of over 200,000 samples and over 2 million components, including, among others, clinical notes, clinical messaging, medications, orders, ICD9 codes, tumor registry, and death index information. In terms of genomics, BioVU contains nearly 200,000 subjects with DNA samples, including dense, GWAS-level genotyping. He discussed the use of BioVU for phenome-wide association studies (PheWAS) to discover all phenotypes associated with a particular genotype, a reverse-engineering approach to GWAS studies[21-23]. As a result of large-scale biorepositories, the Electronic Medical Records and Genomics (eMERGE) network began at 10 sites with a goal to perform GWAS using electronic medical record (EMR) derived phenotypes and to initiate implementation of actionable variants into the EMR[24].

Santosh Kumar (University of Memphis) closed out the Medical Informatics Session with “Designing Sensor-triggered Just-in-Time Mobile Health Interventions – Challenges and Opportunities.” This presentation centered on the National Institutes of Health (NIH) Big Data to Knowledge (BD2K) initiative. Dr. Kumar serves as the director of the MD2K Center of Excellence – NIH Big Data to Knowledge Mobile Data to Knowledge – Mobile Measurement of Exposure, Risk, and Outcomes at the University of Memphis. During his presentation, Dr. Kumar discussed how recent advances in wearable sensors and mobile technology have allowed for the measurement of dynamic changes in an individual's health state as well as physical, biological, behavioral, social, and environmental factors that contribute to health and disease risk[25,26]. He discussed opportunities in detection, prediction, and adaptation, including data measurement in sensor and platform design; prediction of adverse events on a personalized basis; and treatment options in terms of engaging individuals and suggesting non-adverse adaptations. Technologies that can trigger just-in-time mHealth interventions include smartwatches and chestbands which help to measure electrocardiograms, GSR, respiration, and temperature; smartphones which can measure location via GPS, acceleration, and allow for self-reporting; and other EasySense (contactless) sensors such as eyeglass and autosense sensors. The two key contributions of MD2K are in the area of data science research for the development of sensors, and in developing approaches for knowledge discovery.

## Session III: epigenetics

Amelie Baud (European Bioinformatics Institute) led off the final scientific session on Sunday morning with “Leveraging genetic variation in the social partners to study effects of the social environment on phenotypes of biomedical relevance.” In this presentation, Dr. Baud expanded upon work that shows phenotypes of individuals are socially affected by the genetics of the individuals in which they interact. The example used considered aggressive behavior and growth rate of pigs relative to the genetics of their pen-mates[27-30]. Based on this study, Dr. Baud's group has studied whether or not the behavior of a lab mouse or rat is affected by the genotypes of its cage-mates. The results indicate that some phenotypes, including anxiety, growth, and wound healing are dependent on cage-mate genotype, but others including general locomotor activity or helplessness, are not. Their research suggests indirect genetic effects on gene expression likely lead to the differences in phenotype expression.

Alicia Smith (Emory University) followed with “Leveraging epigenome-wide data for human studies of complex traits.” Dr. Smith opened with an introduction of how epigenetic changes are measured, showing there are many experimental design issues to consider in methylation studies, including identification and control of sources of biological and technical variation that can influence interpretation. Some of the work presented included use of umbilical cord blood samples from the CANDLE project to show that DNA methylation varies with gestational age[31]; an illustration that methylation changes across the lifespan[32], differs by race[33], is influenced by medication exposure, has sex-specific effects (such as hormonal influence), varies by cell type, and varies by genotype[34]. As a result of all of these influences, Dr. Smith discussed the use of proxy tissues to create a controlled epigenetic environment[35].

Genetic-epigenetic correlations were described in detail, as part of a research effort involving analysis of 222,888 common SNPS and 20,093 CpG sites, excluding methylation probes that contained SNPs[36]. From this study, 2000 meQTLs in each of 7 cohorts were enriched in GWAS studies and miRNA binding sites. Interestingly, 40-70% of meQTLs overlap between African American and Caucasian cohorts, while 44-51% of meQTLs overlap at birth and in adulthood; 19-37% of meQTLs are detected in both blood and brain cohorts; and only 36-72% are common between different brain tissues in the same person.

Michael Kobor (University of British Columbia) closed the scientific session with “Epigenetic variation in human health and disease.” He built upon the research presented by Dr. Smith, showing that epigenetic imprints vary widely, and they are largely based on diet, environment, and quality of maternal care. Examples of issues with DNA methylation include a higher correlation within than between tissues[37], showing that DNA methylation is tissue specific. Dr. Kobor pointed out that individual tissue type is most important for determining methylation status, followed by cell type. Dr. Kobor also showed the difficulty in linking epigenetic and disease factors, since DNA methylation and gene expression are not tightly linked across individuals[38] and sites with high variability in methylation are largely not correlated with gene expression. Most of the research Dr. Kobor presented was based on “Early Life Experiences Getting Under the Skin” where he discussed the effect of early life socio-economic status and parental stress level on childhood temperament, behavior, and trajectories[38-40], and the effect of maternal warmth on resilience to a pro-inflammatory phenotype[41].

## Future plans

The 2016 UT KBRIN Bioinformatics Summit is scheduled for April 8-10 at Lake Barkley State Park in Cadiz, Kentucky and the 2017 Summit is scheduled for April 21-23 at Montgomery Bell State Park in Tennessee. Sessions will focus more on bioinformatics research in Kentucky and Tennessee to help forge more collaborative efforts. Plans are to include more Tennessee and Kentucky speakers, in terms of intermediate length (20-30 minute) presentations and short (10 minute) presentations that complement plenary session topics.
